# Supplementary material for: Theoretical Investigation of Interconversion Pathways and Intermediates in Hydride/Silyl Exchange of Niobocene Hydride–Silyl Complexes: A DFT Study Incorporating Conformational Search and Interaction Region Indicator (IRI) Analysis
Source: Molecules. 2024 Oct 26;29(21):5075. doi: 10.3390/molecules29215075 (PMC11547935; doi:10.3390/molecules29215075)
Supplement: Supplementary file 1 [file molecules-29-05075-s001.zip › molecules-3251727-supplementary.pdf]

Theoretical Investigation of Interconversion Pathways and Intermediates in Hydride/Silyl Exchange of Niobocene Hydride–Silyl Complexes: A DFT Study Incorporating Conformational Search and Interaction Region Indicator
(IRI) Analysis

Dapeng Zhang and Naoki Kishimoto *

Department of Chemistry, Graduate School of Science, Tohoku University, 6-3, Aoba, Aramaki, Aoba-ku,
Sendai 980-8578, Japan; zhang.dapeng.c5@tohoku.ac.jp
* Correspondence: kishimoto@tohoku.ac.jp; Tel.: +81-22-795-6576

Supporting Information

Table S1. Structural parameters of complexes **3**–**8**, including bond distances (Å) and bond angles (°).

Complex	Nb-H(coordinated)-Si angle	Nb-H(coordinated) distance	H(coordinated)-Si distance	Cl-Si distances	H(uncoordinated)-Nb distances	Nb-Cl distances
**3**	164.42	1.87	1.60	2.22	1.74	4.09
				2.32	1.72	3.63
**4**	164.24	1.89	1.59	2.28	1.74	3.94
				2.28	1.74	3.93
**5**	164.44	1.87	1.60	2.22	1.72	4.09
				2.32	1.74	3.64
Complex	Nb-Si distance				H(uncoordinated)-Nb distances	Cl-Si distance
**6**	2.51				1.79	2.27
					1.83	
**7**	2.50				1.79	2.22
					1.77	
**8**	2.53				1.80	2.19
					1.79	

Table S2. Molecular orbital analysis of complexes **3-8** and HOMO-LUMO energy gaps (eV).

Complex	HOMO	LUMO	HOMO-LUMO gap
**3**	-6.306353	-2.376038	3.930315
**4**	-6.409488	-2.058214	4.351274
**5**	-6.306942	-2.376226	3.930715

Complex	HOMO-alpha	LUMO-alpha	HOMO-LUMO gap (alpha)	HOMO-beta	LUMO-beta	HOMO-LUMO gap (beta)
**6**	0.805655	2.985648	2.179993	-1.259018	2.659119	3.918138
**7**	0.865326	3.163208	2.297881	-1.028798	2.479946	3.508744
**8**	0.915811	3.008835	2.093024	-1.073838	2.728059	3.801897

Figure S1. Molecular orbital HOMO-LUMO diagram of complexes **3-8**.

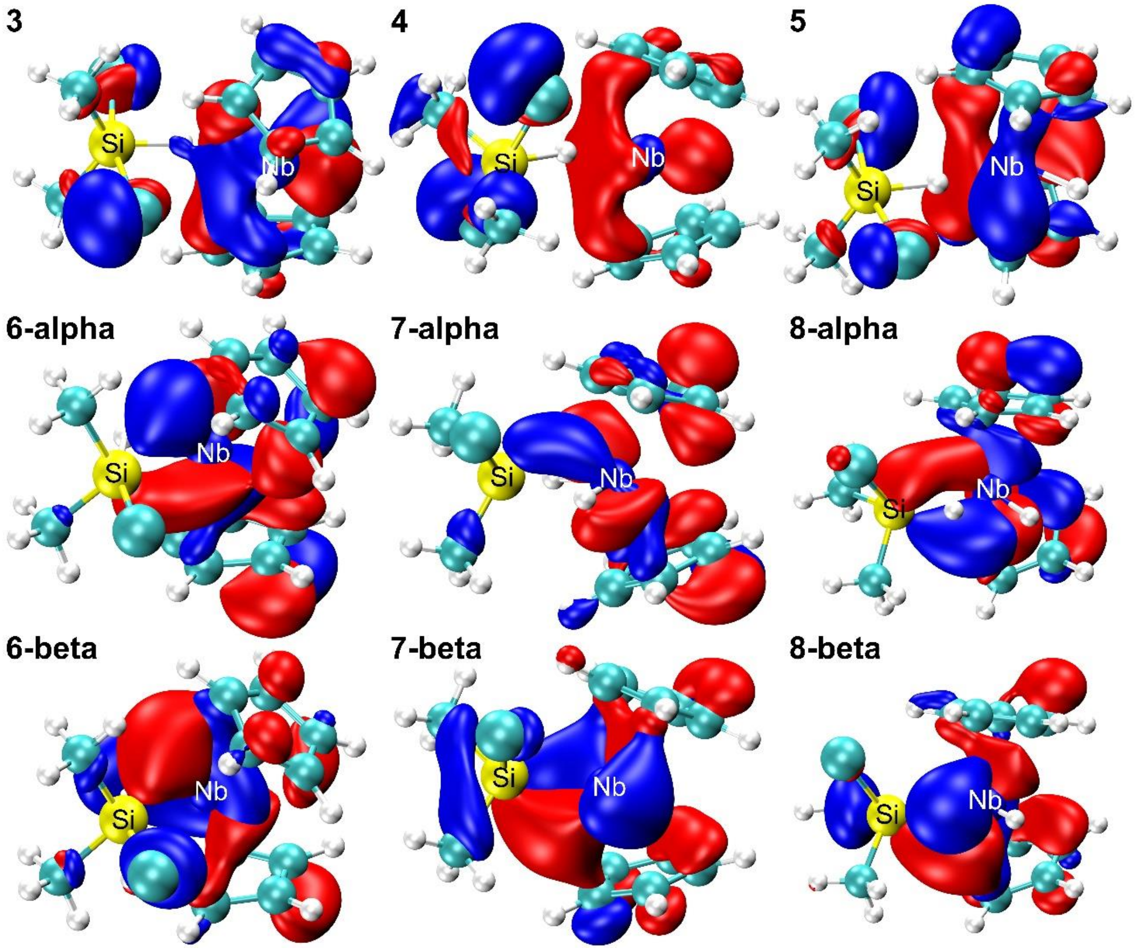

Table S3. Structural parameters of complexes **10**–**15**, including bond distances (Å) and bond angles (°).

Complex	Nb-H(coordinated)-Si angle	Nb-H(coordinated) distance	H(coordinated)-Si distance	Cl-Si distances	H(uncoordinated)-Nb distances	Nb-Cl distances
**10**	159.23	1.88	1.63	2.26	1.72	3.95
				2.27	1.75	3.69
**11**	177.22	1.90	1.60	2.27	1.74	3.95
				2.31	1.74	3.94
**12**	159.84	1.88	1.62	2.23	1.75	3.96
				2.31	1.72	3.70

Complex	Nb-Si distance	H(uncoordinated)-Nb distances	Cl-Si distance
**13**	2.52	1.79	2.28
		1.84	
**14**	2.50	1.79	2.23
		1.77	
**15**	2.54	1.80	2.21
		1.78	

Table S4. Molecular orbital analysis of complexes **10-15** and HOMO-LUMO energy gaps (eV).

Complex	HOMO	LUMO	HOMO-LUMO gap
**10**	-6.293351	-2.282532	4.010819
**11**	-6.285540	-2.072300	4.213240
**12**	-6.308049	-2.280480	4.027569

Complex	HOMO-alpha	LUMO-alpha	HOMO-LUMO gap (alpha)	HOMO-beta	LUMO-beta	HOMO-LUMO gap (beta)
**13**	0.489082	2.791670	2.302588	-1.299754	2.352482	3.652237
**14**	0.657152	3.019766	2.362614	-1.096579	2.257133	3.353712
**15**	0.750699	2.818866	2.068167	-1.139584	2.555684	3.695267

Figure S2. Molecular orbital HOMO-LUMO diagram of complexes **10-15**.

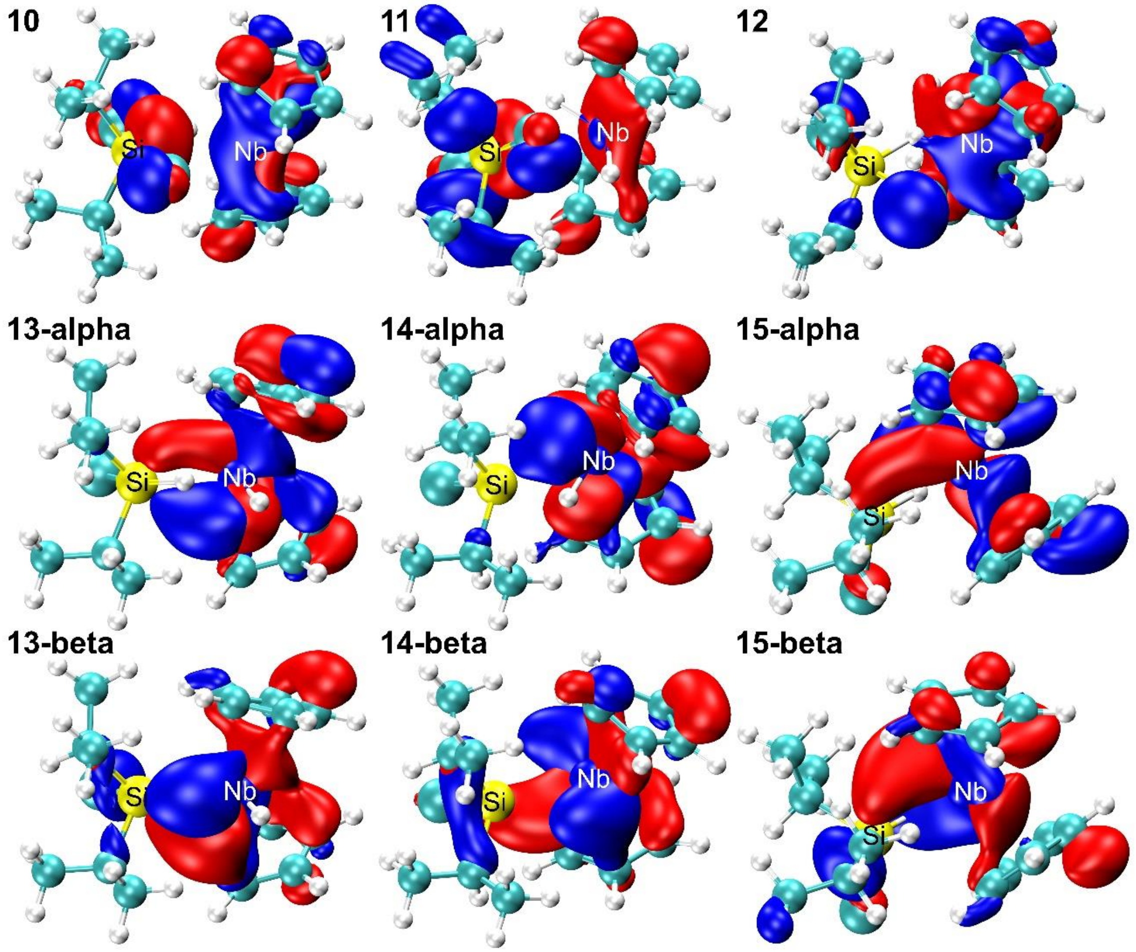

Table S5. Structural parameters of complexes **17**–**24**, including bond distances (Å) and bond angles (°).

Complex	Nb-H(coordinated)-Si angle	Nb-H(coordinated) distance	H(coordinated)-Si distance	Cl-Si distances	H(uncoordinated)-Nb distances	Nb-Cl distances
**17**	153.00	1.90	1.60	2.28	1.74	3.89
				2.26	1.74	3.90
**18**	152.88	1.90	1.60	2.28	1.74	3.89
				2.26	1.74	3.90
**19**	153.05	1.90	1.60	2.28	1.74	3.89
				2.26	1.74	3.90
**20**	152.85	1.90	1.60	2.28	1.74	3.89
				2.26	1.74	3.90
Complex	Nb-Si distance				H(uncoordinated)-Nb distances	Cl-Si distance
**21**	2.49				1.78	2.21
					1.78	
**22**	2.49				1.79	2.21
					1.77	
**23**	2.49				1.78	2.21
					1.78	
**24**	2.49				1.79	2.21
					1.77	

Table S6. Molecular orbital analysis of complexes **17-24** and HOMO-LUMO energy gaps (eV).

Complex	HOMO	LUMO	HOMO-LUMO gap
**17**	-6.335669	-2.055237	4.280432
**18**	-6.334050	-2.055207	4.278843
**19**	-6.333074	-2.055479	4.277595
**20**	-6.337742	-2.055039	4.282703

Complex	HOMO-alpha	LUMO-alpha	HOMO-LUMO gap (alpha)	HOMO-beta	LUMO-beta	HOMO-LUMO gap (beta)
**21**	0.738771	2.443915	1.705143	-1.299567	2.098279	3.397845
**22**	0.713161	2.420751	1.707591	-1.297939	2.222880	3.520818
**23**	0.740960	2.440366	1.699406	-1.307710	2.098191	3.405901
**24**	0.711960	2.420139	1.708179	-1.301443	2.222013	3.523456

Figure S3. Molecular orbital HOMO-LUMO diagram of complexes **17-24**.

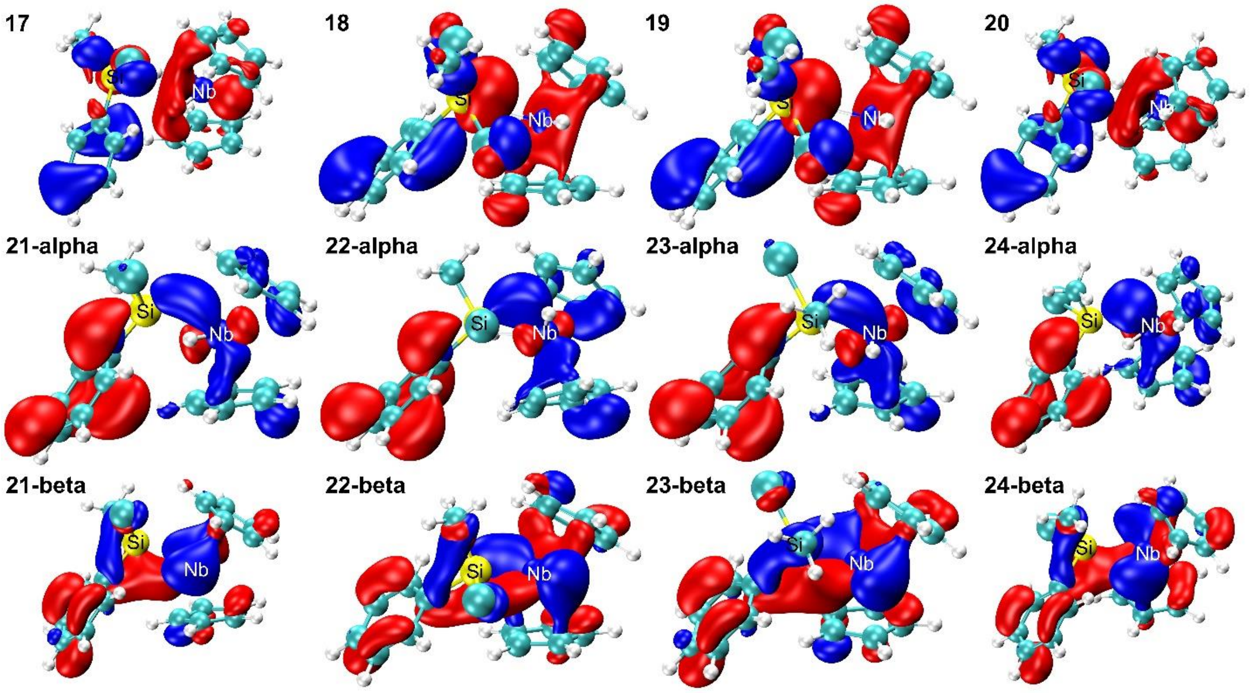

Structural Coordinates of the Complexes

Complex 1

Nb	19.156894013254	-8.254508336499	-0.268715896008
C	19.437292656588	-10.549103848268	-0.854458521815
C	18.042527269703	-10.276640354765	-0.921456077959
C	19.849332180264	-10.418944675772	0.501052720926
C	17.603346269206	-9.941460018773	0.381016699819
C	18.721536616664	-10.029434766504	1.261388399928
C	18.158121205710	-6.106304334126	-0.649801488232
C	17.695785427460	-6.583384791729	0.599323773511
C	19.565482493982	-5.922123898747	-0.552197941400
C	18.813453903064	-6.672219727223	1.480256046148
C	19.964139241045	-6.249914411428	0.773625171265
H	18.467467182640	-8.131152517918	-1.867130283507
H	20.875213511044	-8.321354580048	0.028331116088
H	17.555015764417	-5.914398666004	-1.520673780599
H	16.674249476996	-6.828152403989	0.845065513391
H	18.785245237699	-6.995938375932	2.508957094274
H	20.214157922114	-5.580248177987	-1.340721067451
H	20.964349173619	-6.185280295350	1.166526954403
H	17.432426907050	-10.320307408408	-1.807371410843
H	20.068574603059	-10.820724785091	-1.683393308639
H	20.843291976285	-10.588943599806	0.878047561813
H	18.708073258026	-9.841100503520	2.323538653956
H	16.596039256863	-9.675165148464	0.660736921336
H	20.247585503248	-8.194497223656	-1.647979260405

Complex 2

Si	-0.315555554648	-0.249310549045	-1.183845319626
C	0.257144484258	0.740039231434	-2.647325476154
C	0.217752965687	0.433617273080	0.458651350264
Cl	0.409423586698	-2.174197848199	-1.364517401668
Cl	-2.377754654889	-0.346528834959	-1.219555466888
H	1.348782817433	0.786365161707	-2.671137316343
H	-0.088829341454	0.287167048988	-3.578053399167
H	1.308318959595	0.472438786949	0.518217104028
H	-0.174614836073	1.444400088437	0.595579113728
H	-0.149730824325	-0.191953542166	1.273921398138
H	-0.133495512273	1.759301743771	-2.593547686315

Complex 3

Nb	19.766754572566	-8.109365823549	-0.381674122095
C	20.200288610196	-10.046936170469	-1.690235196751
C	18.794622405935	-9.996654884498	-1.465405780139

C	20.829743973895	-10.275654186911	-0.434750987509
C	18.574472193316	-10.139947602965	-0.070592423396
C	19.838654317218	-10.314844203729	0.563983535957
C	18.258758089960	-6.291179106990	-0.065858761895
C	18.145558654705	-7.145993017567	1.061504155348
C	19.579927849615	-5.758590069946	-0.075613662532
C	19.382498707757	-7.115827975943	1.768998125183
C	20.251429067315	-6.250038176443	1.078504174704
H	18.733123017388	-7.759362613636	-1.705066954284
H	21.612606107033	-7.805065685091	-0.486657512769
H	17.481146113101	-6.069121589062	-0.776683596142
H	17.265202855579	-7.699925371234	1.346913901381
H	19.633443263002	-7.671409645802	2.658034237556
H	19.987799565589	-5.082487810589	-0.808363604699
H	21.269389439723	-6.045062743806	1.362961370914
H	18.035773061782	-9.887820671655	-2.221424540788
H	20.696048316490	-9.957318266914	-2.642404154752
H	21.888070050542	-10.356735232954	-0.254392883643
H	20.022463660270	-10.429480753172	1.619997953043
H	17.613343354233	-10.143731066822	0.418859410243
H	20.316554325661	-7.455221269431	-1.902213804503
Si	23.173991642581	-7.703985821816	-0.173513683994
C	24.285290008166	-9.162211872204	-0.565079900694
C	23.845454323065	-6.115805718460	0.561734543929
Cl	22.561513408174	-8.562056179532	1.897884592001
Cl	23.209956265297	-6.931412190469	-2.255052897144
H	24.545735111500	-9.741829149565	0.318906612652
H	23.814441145510	-9.812398283481	-1.306228838099
H	24.030737179325	-6.192738769277	1.631777628963
H	24.772579901718	-5.878683340301	0.033572942473
H	23.162167112078	-5.286356902237	0.364643105606
H	25.187863139662	-8.766016163518	-1.037492424022

Complex 4

Nb	20.034383665158	-8.194612856329	-1.646169808858
C	19.915405245213	-10.515005191522	-2.176838409920
C	18.630725598653	-9.967022919154	-2.438606471242
C	20.175540943344	-10.416237293823	-0.785882110429
C	18.106941145593	-9.495450805667	-1.210901117190
C	19.064032533988	-9.769874418002	-0.187344337528
C	19.549328351461	-5.917138484341	-2.182543081345
C	18.648157013301	-6.345153623329	-1.173270850090
C	20.855924287826	-5.953288791601	-1.633797008517
C	19.403361293546	-6.613665262134	0.008141897116
C	20.766630883222	-6.364381215970	-0.276695591451
H	19.620341658287	-7.990109307268	-3.325678837029
H	21.574687171537	-8.522226616360	-0.904506778183

H	19.307046175940	-5.629517202218	-3.191172073875
H	17.577257919515	-6.423463809270	-1.271961842746
H	19.002793297442	-6.940242008235	0.954610089379
H	21.757504123141	-5.735764006250	-2.183425462942
H	21.591794875077	-6.465303071754	0.408030926448
H	18.146816464071	-9.921235794185	-3.399535239366
H	20.607221366473	-10.913644767149	-2.901268326019
H	21.070705995308	-10.760821402804	-0.297415753977
H	18.949814490188	-9.549130536446	0.861860359177
H	17.147052434084	-9.024470795983	-1.070541366170
H	21.419481268056	-8.440221469555	-2.906994066986
Si	22.822182076897	-8.452324762962	-3.658234318127
C	22.902057320206	-9.387561655400	-5.279567901908
C	24.237986219169	-7.526467674759	-2.856304329309
Cl	23.231990209172	-10.333296004183	-2.436336979797
Cl	21.966297399202	-6.603909038814	-4.691699365386
H	23.578262516977	-10.238827066184	-5.207355761473
H	21.902011112848	-9.762503758911	-5.516853146180
H	25.112727561799	-8.176450855437	-2.801731618551
H	24.474154804980	-6.607809948361	-3.391797672421
H	23.958767349225	-7.279691968501	-1.828079703114
H	23.198539379059	-8.716759477140	-6.086370201990

Complex 5

Nb	19.615789930265	-8.065103747047	-1.232178551058
C	19.748506490723	-10.318823458647	-1.978353247208
C	18.614557519106	-9.758539466977	-2.630360055024
C	19.469910974402	-10.353443670549	-0.581878011985
C	17.666234941515	-9.400832836076	-1.653733825690
C	18.193567771390	-9.766020784193	-0.380921108969
C	19.389647588954	-5.668398079955	-1.405117393807
C	18.279059612456	-6.148266064378	-0.685565921420
C	20.570029383093	-5.961517156718	-0.666806042526
C	18.760929648254	-6.719696666750	0.528002102833
C	20.173696890287	-6.585493817180	0.550906752016
H	20.015644801981	-7.610079508939	-3.005375352457
H	20.837308163645	-8.616091504556	-0.161338487097
H	19.339952158327	-5.204647414382	-2.375506068545
H	17.255155960485	-6.107873711094	-1.020286092842
H	18.156196139035	-7.154349836063	1.308204122178
H	21.578994846509	-5.727007639948	-0.962422332388
H	20.828402807114	-6.884072514268	1.351836368097
H	18.504881447686	-9.583796990797	-3.687029772446
H	20.643598625227	-10.678447675619	-2.457851593595
H	20.106724235172	-10.768003896147	0.180922613202
H	17.698285436084	-9.644028484077	0.569423317043
H	16.725064227266	-8.912560215622	-1.848434835476

H	21.292912813726	-8.246335002904	-1.675713120624
Si	19.931728014669	-7.142770920058	-4.529057630659
C	19.594151877140	-8.417841757257	-5.861351084541
C	20.192724732750	-5.331407627430	-4.934999103446
Cl	22.105018599245	-7.591324745323	-4.445832616747
Cl	17.675503533366	-6.849149161993	-4.056679820729
H	20.276397115710	-8.211314681433	-6.690012375356
H	19.844827823627	-9.419195961503	-5.503334923871
H	20.846193996132	-5.283943785426	-5.809900629143
H	19.259767804181	-4.806124825887	-5.132401689906
H	20.731394416696	-4.833414598067	-4.125298325369
H	18.562600753715	-8.399976262786	-6.208928212493

Complex 6

Nb	19.039889648876	-8.334896908507	-0.230885997494
C	17.475096728375	-9.210540737696	-1.879365426234
C	16.695282297565	-9.252531500560	-0.688658010849
C	18.556214995872	-10.119979551981	-1.738278442905
C	17.329182356444	-10.133891764349	0.205847146322
C	18.483338507625	-10.676333964713	-0.430229675115
C	18.449995321242	-6.066886020541	0.637189174122
C	17.411870424939	-6.758561399468	1.332053788966
C	19.696724545316	-6.500572168848	1.154850157934
C	18.016616044208	-7.691465736860	2.179009577960
C	19.434774943940	-7.559602680697	2.068691707706
H	19.148606194941	-7.258173657546	-1.654687944171
H	20.749382589431	-8.064126674821	-0.822083509296
H	18.312408288194	-5.278061276141	-0.085457514067
H	16.353665976425	-6.662341763214	1.138477842020
H	17.509849344308	-8.420294300357	2.794201365494
H	20.666689833834	-6.111479770605	0.891851294042
H	20.159261457584	-8.083447744492	2.672279675745
H	15.818714275267	-8.656925861166	-0.485680593019
H	17.261472159509	-8.618451185463	-2.754404537387
H	19.308064051596	-10.336051711192	-2.480600340194
H	19.142479623334	-11.417134529080	-0.005845289845
H	17.014178927382	-10.356146778624	1.213765471086
Si	21.248713134803	-9.400943621308	0.311003768844
C	22.686236630575	-8.372182528021	1.024412223374
C	22.048749810531	-10.511333684563	-1.013743102616
Cl	21.217986996859	-10.947648909044	1.973129644512
H	21.363586629882	-11.301654093870	-1.333122376122
H	23.509330769035	-9.024125241823	1.329562915447
H	22.377689773671	-7.795008254589	1.900371158914
H	23.053384130166	-7.667140739111	0.271284317551
H	22.951713762196	-10.991686791902	-0.625737452575
H	22.312211276089	-9.918257678856	-1.895161448147

Complex 7

Nb	19.214239462436	-8.360108033712	-0.070811994072
C	17.724679123340	-9.583946826973	-1.571453903317
C	16.879087946929	-9.466586817296	-0.429620811801
C	18.825756857856	-10.415077302712	-1.220212699677
C	17.470300778728	-10.171289061594	0.628701816111
C	18.695816497867	-10.730655349320	0.161132058081
C	18.611944896820	-6.028894536123	0.334134442965
C	17.577726726579	-6.643600474534	1.099415311301
C	19.859802491025	-6.327875389810	0.952846956671
C	18.174466922535	-7.358981469626	2.145160232282
C	19.587702824202	-7.200195633192	2.044559741194
H	19.499458816571	-7.522038947368	-1.624653529387
H	20.689706460989	-9.056576373228	0.621774000441
H	18.469474204765	-5.383936435085	-0.518031934564
H	16.523618584567	-6.617073503176	0.866039414890
H	17.663126943473	-7.984418373026	2.861781475819
H	20.826211893189	-5.949565140357	0.662642019414
H	20.319548704024	-7.605344598391	2.724405829479
H	15.972605205254	-8.881828408002	-0.377169227103
H	17.528449666749	-9.179272360262	-2.551690153712
H	19.593101883788	-10.788089393364	-1.879024325636
H	19.375316191398	-11.349201557523	0.724494050723
H	17.101035682565	-10.224388768005	1.642008626883
Si	21.298594401418	-8.582561352344	-1.427016929113
C	22.676942148340	-7.303760279950	-1.108031184702
C	21.229463404638	-8.662293489234	-3.339570024441
Cl	22.475559237798	-10.449010573943	-1.159786433298
H	20.513418415234	-9.416951537102	-3.680240527418
H	23.570538573250	-7.516973648738	-1.703814001322
H	22.319510631153	-6.300580906011	-1.367000385682
H	20.896526680757	-7.694880000160	-3.731988816199
H	22.960105593335	-7.299671022947	-0.052084409581
H	22.205235878433	-8.903931086896	-3.771541995218

Complex 8

Nb	19.157550044379	-8.296525578589	-0.093268689099
C	17.997556426606	-9.953198257334	-1.483489401508
C	16.980601781804	-9.761112770273	-0.498476043653
C	19.102329941194	-10.575225208297	-0.841775296989
C	17.446020567541	-10.249321650043	0.728816462303
C	18.791536026226	-10.683139895022	0.537362539147
C	18.284949668146	-6.036636760929	-0.116180198478
C	17.273790111728	-6.734207288174	0.607783046434
C	19.474417666076	-6.057274283456	0.667837248001
C	17.825244663090	-7.190041189855	1.816714383611

C	19.197786985315	-6.808765536836	1.839530627470
H	20.680381926048	-8.496558479478	-1.040968500715
H	20.595320486643	-8.734641456272	0.869961661844
H	18.143968457860	-5.503087953074	-1.042483423165
H	16.262825175351	-6.899766850436	0.267235700841
H	17.324691469884	-7.790805739890	2.560090110161
H	20.413942938386	-5.593896100823	0.412426484995
H	19.897917978064	-7.018093032522	2.631661713106
H	16.017941074916	-9.304732201171	-0.679108319435
H	17.898744926216	-9.794286390218	-2.545448466684
H	20.017580557834	-10.874832940555	-1.324210636587
H	19.434367809208	-11.107319531036	1.292612101525
H	16.934607550365	-10.181916131437	1.677530527183
Si	19.795894587350	-7.572315422502	-2.429808375290
C	21.113469612617	-6.203667934615	-2.557114819224
C	18.463038280566	-6.938761918324	-3.671899916176
Cl	20.613990902277	-9.118128363557	-3.743145499814
H	17.684001197049	-7.691376471301	-3.826634194721
H	21.380780468788	-5.999916068216	-3.599088442926
H	20.729964478597	-5.273758071189	-2.120442052590
H	22.016895661945	-6.482405958936	-2.009863755345
H	18.894488349398	-6.693927629803	-4.648569794957
H	17.974550668532	-6.038833125849	-3.279094849260

Complex 9

Si	-0.978539392204	0.855028731163	-0.390875839172
Cl	-2.597380390827	-0.230259166747	-1.092442142142
Cl	-0.858274717896	0.539635126646	1.653768371010
C	-1.327048776913	2.679498818206	-0.684870791691
C	-1.727857003315	2.977506375063	-2.139503868314
H	-2.661467878292	2.470633306303	-2.392421347574
C	-2.396993637494	3.221237652179	0.276306307974
H	-2.110694587068	3.096072366681	1.320611260938
H	-2.559517460773	4.286529627388	0.089024788914
H	-1.885854194802	4.052209528726	-2.266613297266
H	-0.378283265197	3.190163434669	-0.470177972314
H	-0.972256033256	2.663643641022	-2.860179094421
H	-3.349732917370	2.707943274819	0.123223591221
C	0.592594829191	0.139473900332	-1.132768504837
C	0.717477187111	0.370392798654	-2.646489237748
H	0.784466511028	1.429867667656	-2.898064009766
C	0.733001522073	-1.354383587487	-0.799624786978
H	-0.061628320363	-1.933089565075	-1.275997282794
H	1.690564331328	-1.730601033033	-1.170657944592
H	1.621372573328	-0.116983488239	-3.023209047386
H	1.405369512907	0.680301733416	-0.629514330964
H	-0.134711090619	-0.054231892498	-3.182962118703

H	0.687474779371	-1.538457249831	0.274661826608

Complex 10

Nb	19.165053693270	-8.586683338752	-0.109566047875
C	17.817749939253	-9.406819337188	-1.895345835677
C	16.980464608123	-9.240772671379	-0.761998577029
C	18.774358693574	-10.415835283573	-1.582823447859
C	17.399119578563	-10.164526773960	0.239771613316
C	18.486959101078	-10.894293628327	-0.276593634409
C	19.200289743132	-6.205892753358	-0.009337070083
C	18.075032294096	-6.650255190830	0.733310008922
C	20.370621170818	-6.680263382759	0.647690137598
C	18.547001798463	-7.369045615147	1.870062297179
C	19.954522321156	-7.363693612416	1.823473820807
H	20.607175051627	-8.665823191393	-1.091078560962
H	20.565025731755	-9.603998470713	0.632068000137
H	19.175165166119	-5.601569153471	-0.900084726924
H	17.042442880361	-6.452525252047	0.493198475626
H	17.944708301353	-7.843632541683	2.628188010291
H	21.390556738509	-6.543391851731	0.329752069441
H	20.603612472870	-7.817879224454	2.552654874574
H	16.153624708958	-8.552745542975	-0.684973170403
H	17.732946969562	-8.882123432817	-2.831740606749
H	19.555899240675	-10.772798987545	-2.231867661153
H	19.038718188630	-11.638132787018	0.270779656143
H	16.981974346951	-10.280995651331	1.226882907883
Si	21.601729557998	-10.180835283756	1.744601158782
C	22.241441426085	-11.882133685296	1.193026243240
C	22.381293562015	-9.386640644968	3.316617774495
Cl	23.052198779602	-9.045081666387	0.443589770706
C	21.589575126896	-9.356320393068	4.624360787606
H	21.372475521042	-10.362851467094	4.983829534633
H	22.178127964846	-8.839853664321	5.390174442775
C	23.739189364882	-10.081386114415	3.548368758404
H	24.279346328135	-9.569382811285	4.350505759634
H	24.362275252703	-10.059975208389	2.654195672624
H	22.609216265784	-8.358192248950	3.019003165782
C	21.883264051469	-13.059356572062	2.103335243617
H	20.819533544985	-13.294102611466	2.046427701753
H	22.446871225486	-13.947470581441	1.798562300389
C	21.915568931219	-12.199012378869	-0.272463260348
H	22.509675440181	-13.051620131676	-0.615342903794
H	20.865015181007	-12.474594863727	-0.390318637794
H	22.129308687738	-11.349909349906	-0.923542406268
H	22.113375303539	-12.857168517668	3.151176770514
H	23.324117983172	-11.718494995004	1.249051136770
H	23.602204616715	-11.120839725447	3.856711570214

H	20.632725080673	-8.840894132489	4.526498563164
H	19.345498082885	-7.776960505887	-1.610934409777
Cl	19.677606472096	-10.841879191587	2.761174589972

Complex 11

Nb	19.181744194738	-8.328117835602	-0.159183108454
C	17.853281952241	-9.742049215636	-1.569664860077
C	17.196164263518	-9.613054805106	-0.322917561711
C	19.089613902938	-10.401537808990	-1.338292769873
C	18.024234895585	-10.206344614112	0.677314894923
C	19.188738113264	-10.708838183719	0.042120159110
C	18.710379475474	-5.987602753517	-0.073559320537
C	17.732212065169	-6.673142893541	0.690824887310
C	19.964574252916	-6.190627840716	0.555766369394
C	18.386478246885	-7.276725493532	1.807216632774
C	19.765931542035	-6.968988039289	1.726589580153
H	18.982657683985	-7.640252304561	-1.747534999396
H	20.611180420995	-8.916076417152	0.642588498627
H	18.551727725536	-5.421347256297	-0.975140471555
H	16.675040578732	-6.707978785252	0.481997256157
H	17.909878361726	-7.853422304770	2.583661208951
H	20.905306420672	-5.835527233438	0.169363656116
H	20.531424057944	-7.267718874378	2.422598657374
H	16.232531413960	-9.158077351736	-0.158399324712
H	17.487155257948	-9.403013543876	-2.524093249198
H	19.857739030640	-10.611206796156	-2.064624201834
H	20.014711866377	-11.220287579179	0.505627129595
H	17.791238685621	-10.285020514301	1.727098132582
Si	22.104772165605	-8.088433415284	-2.066516567500
C	23.739367284016	-7.632270771938	-1.203640173417
C	21.941107223749	-8.563678242308	-3.902952728861
Cl	22.371569735195	-10.286997830967	-1.401642279277
C	20.496856873332	-8.473798544856	-4.409983171814
H	20.123261591586	-7.450984485196	-4.338730497900
H	20.448955304769	-8.785963548258	-5.458364864575
C	22.877261443105	-7.736444471304	-4.795570137201
H	22.770301028345	-8.059295604764	-5.836572179404
H	23.925032336231	-7.867281183934	-4.515509363227
H	22.258197978338	-9.607640321135	-3.946442601317
C	24.468994384487	-6.385966712785	-1.712532674810
H	23.916581123955	-5.477292044332	-1.473597005776
H	25.453727849107	-6.325340987993	-1.236251940094
C	23.542850675471	-7.554807657444	0.317669143123
H	24.510730862124	-7.456568002696	0.819251632417
H	22.948967016205	-6.676154081527	0.584671847923
H	23.044225864166	-8.445163460819	0.704307516184
H	24.618496127321	-6.403793103758	-2.792976812305

H	24.360665618678	-8.510264306204	-1.407653838250
H	22.637876950458	-6.673386928773	-4.742981472332
H	19.823683025370	-9.117211529302	-3.839387406244
H	20.793022708142	-8.189925488597	-1.161099101838
Cl	21.410680161326	-5.950446090990	-2.396595979212

Complex 12

Nb	19.099827338137	-8.355909822996	-0.098317883896
C	18.290867721460	-9.422950178811	-2.116408332625
C	17.204828296189	-9.307454166506	-1.226142448486
C	19.280439803055	-10.253799558387	-1.526032085213
C	17.498187010635	-10.108254035452	-0.083696745317
C	18.770320474027	-10.707908287225	-0.276316799710
C	18.978509689741	-6.046307457192	0.585111000149
C	17.747889950149	-6.652600533655	0.903103675151
C	20.003717225528	-6.682248492762	1.334853968430
C	17.998655678651	-7.657660835328	1.882473278190
C	19.389446015802	-7.665307975681	2.163486060523
H	20.825218165063	-8.456937376972	-0.355439914487
H	20.148717484278	-9.328548182618	0.848743724923
H	19.107173945266	-5.277674256920	-0.157193879025
H	16.797811358893	-6.404303727555	0.458667472331
H	17.257885090145	-8.285761675002	2.351199187616
H	21.053637239513	-6.445227573461	1.303800888574
H	19.889437465266	-8.285299145450	2.888037936380
H	16.321772994220	-8.709970838641	-1.386648351131
H	18.364206046868	-8.945084258585	-3.078514230440
H	20.234018289153	-10.499883571879	-1.961855526251
H	19.254730321387	-11.399993392109	0.391291017165
H	16.853758901811	-10.255189211329	0.768381892010
Si	19.811268608925	-6.690405729785	-3.031914801681
C	20.857218693179	-5.103823480723	-2.902783487076
C	19.023925345288	-7.203863359510	-4.716403539040
Cl	21.581282253421	-8.035386283543	-3.173337638016
C	18.958278170693	-5.941569992558	-5.597995409443
H	19.958466753510	-5.569401466894	-5.835563335022
H	18.464176335966	-6.182012623291	-6.544401996420
C	19.646045900168	-8.355752166963	-5.506966358938
H	19.041494946992	-8.543196167072	-6.401074160666
H	19.698213024544	-9.280765628521	-4.930686688527
H	17.988538352407	-7.463034645110	-4.476532004122
C	20.137043358850	-3.760997320714	-3.048355831627
H	19.465458138732	-3.580703724575	-2.207553148385
H	20.875210290143	-2.952239147778	-3.072923634163
C	21.742353825724	-5.088840678421	-1.646312339827
H	22.510334081120	-4.315160077570	-1.740489945656
H	21.151644233837	-4.845985088128	-0.760025680709

H	22.236749590308	-6.046176660639	-1.482477774166
H	19.540477200084	-3.701250964455	-3.957784152625
H	21.527002944200	-5.235754696672	-3.763076542755
H	20.661959327878	-8.120843386017	-5.825195057428
H	18.392152715152	-5.144812766643	-5.115797807000
H	19.728243223885	-7.336272609191	-1.545433507323
Cl	17.758563439768	-5.819571960744	-2.429350884309

Complex 13

Nb	19.057791054766	-8.349762187059	-0.186773738699
C	17.517033747570	-9.276299952590	-1.829769286903
C	16.738232901293	-9.281704533935	-0.639832594333
C	18.595833184004	-10.183037436123	-1.659702835310
C	17.368999792306	-10.140446805143	0.280558861867
C	18.520323112688	-10.703620672619	-0.338786499582
C	18.430131004883	-6.037045390833	0.570453240286
C	17.325685557419	-6.691472303515	1.193926147984
C	19.616679591045	-6.473126490325	1.207295728441
C	17.835419873074	-7.595377549076	2.128376134144
C	19.259726528497	-7.498221062048	2.127015362353
H	19.181546462144	-7.327887121829	-1.645372685283
H	20.781668189323	-7.925165442119	-0.657766761140
H	18.369078714910	-5.270689940490	-0.185844640409
H	16.289306249274	-6.579465189610	0.911223336340
H	17.261623045468	-8.292331669645	2.721172486649
H	20.609757124999	-6.102275387090	1.017673598983
H	19.923581362043	-8.012210226066	2.801783492556
H	15.862260770191	-8.680260606499	-0.454718921392
H	17.305148136266	-8.709712519064	-2.721865533255
H	19.340921144582	-10.430524751902	-2.397944661859
H	19.186603482532	-11.423209530079	0.109864963981
H	17.051326566305	-10.333813772649	1.293412173633
Si	21.311525470611	-9.360102166607	0.308007145213
C	22.836284156798	-8.455481359007	1.079045276439
C	22.029365111428	-10.386159973570	-1.170853879364
Cl	21.340976104406	-11.066929918735	1.820878952712
C	22.062150255426	-9.617607030815	-2.499267183250
H	22.830775383636	-8.839090522891	-2.481966531178
H	22.300265150219	-10.289376673394	-3.335673740891
C	23.395105101385	-11.028318271014	-0.903076477920
H	23.670851736738	-11.718769086136	-1.711695825876
H	23.396197401032	-11.583022205214	0.037139995327
H	21.304276860850	-11.204115095872	-1.278356339008
C	23.378520398867	-7.342929447592	0.174267754188
H	22.634725572158	-6.555192699470	0.026638058873
H	24.275445199769	-6.881821280515	0.610506804957
C	22.657038506657	-7.937735336686	2.509344829158

H	23.618337562349	-7.605743754777	2.924331212299
H	21.979419562023	-7.081779393795	2.545444180947
H	22.258450089852	-8.717191943561	3.161970465316
H	23.645348601142	-7.718335515539	-0.816141848181
H	23.591469689337	-9.249749281289	1.125852076430
H	24.180375836411	-10.267073167280	-0.848626285682
H	21.112939143321	-9.119496145975	-2.702283309484

Complex 14

Nb	19.232599284341	-8.293575523624	-0.053545833515
C	17.761688142825	-9.496392063287	-1.591069703178
C	16.910997955257	-9.421823070123	-0.449864571476
C	18.868563208100	-10.327680183921	-1.257934809736
C	17.505483505462	-10.148407228041	0.590559963184
C	18.740453249347	-10.678177887324	0.114304136089
C	18.523034595799	-6.002527216226	0.411832267799
C	17.505648545195	-6.686240681549	1.139230106146
C	19.769350854290	-6.260798679258	1.050588335398
C	18.116250635667	-7.406622757609	2.173659039816
C	19.521318970163	-7.180253089710	2.106547025158
H	19.577238950127	-7.400081374802	-1.564193319541
H	20.706109504611	-8.986018773352	0.647593022333
H	18.367497970802	-5.335043343747	-0.420599957974
H	16.455754599800	-6.700479163336	0.887647945585
H	17.621687037806	-8.076771642344	2.860672679435
H	20.717124256249	-5.814326572985	0.803926198764
H	20.259542063569	-7.572867538324	2.787016292725
H	15.997099545984	-8.850416696524	-0.385869168327
H	17.556576927569	-9.078582233523	-2.564011059920
H	19.631786553549	-10.691311625165	-1.925577523022
H	19.425776596335	-11.304265936867	0.661877240596
H	17.133405436628	-10.232695096653	1.600474001071
Si	21.300332624359	-8.544723396700	-1.442025599761
C	22.776431302927	-7.342604659647	-1.145209552692
C	21.196758229416	-8.733198098976	-3.382163647563
Cl	22.423505260666	-10.452860272124	-1.150993262398
C	20.258419450438	-7.743412387640	-4.082568326771
H	20.609403491784	-6.712789393490	-3.970489232922
H	20.201472985490	-7.954085678114	-5.159938396569
C	22.565021303050	-8.731320394410	-4.074914734347
H	22.471164610426	-8.997928847070	-5.136789429403
H	23.248642399651	-9.440084184975	-3.603771397094
H	20.772548587675	-9.736651539691	-3.520576565217
C	22.458475114580	-5.915337334712	-1.608167275911
H	21.552772110374	-5.533676855020	-1.130338569587
H	23.281004308731	-5.227183201572	-1.369804371704
C	23.251932599276	-7.365450892726	0.309895591402

H	24.111738266756	-6.696996505108	0.456044826734
H	22.458387530124	-7.050621020731	0.991083228882
H	23.545385602096	-8.375593815838	0.602745443039
H	22.292509425127	-5.871580238810	-2.687722546547
H	23.597851291708	-7.724341760570	-1.764062320637
H	23.025171257616	-7.738626632025	-4.032440645095
H	19.250771238249	-7.781303061753	-3.667271523245

Complex 15

Nb	19.137220680462	-8.224884101838	-0.086160293289
C	17.976389034392	-9.855687236478	-1.511720822535
C	16.963526699763	-9.681058880087	-0.519634643078
C	19.083963318358	-10.487737596001	-0.886795814819
C	17.431100942030	-10.201496195719	0.694054196570
C	18.776449357167	-10.626528506515	0.490354961338
C	18.325731011201	-5.929965667766	0.091357303403
C	17.286182195199	-6.674844944496	0.717568969172
C	19.495510154995	-6.063482103343	0.887912426405
C	17.797639598057	-7.255572762684	1.891508378485
C	19.177494444650	-6.918577440203	1.975534667714
H	20.657242253425	-8.397208232579	-1.040053927275
H	20.582421030402	-8.721154010703	0.825414815872
H	18.218621262840	-5.310802644133	-0.782508342647
H	16.278551824331	-6.778002406023	0.343501064242
H	17.265709436800	-7.909666430543	2.563893312274
H	20.452487624316	-5.602937096204	0.703272171927
H	19.855706357230	-7.219227486795	2.757242991448
H	15.999531969127	-9.222726659951	-0.688316361435
H	17.869508192610	-9.681035126796	-2.569770060547
H	19.998348504133	-10.774172204379	-1.378620711319
H	19.421811532457	-11.066917359011	1.233993890589
H	16.920901078819	-10.159381361801	1.644480510073
Si	19.800489700012	-7.514581777680	-2.433610859404
C	21.151621910641	-6.160639708727	-2.673991909378
C	18.441548477238	-7.093129670584	-3.782385016684
Cl	20.720574576221	-9.103535949111	-3.661971225321
C	17.268569947568	-6.275180534732	-3.235094259340
H	17.569020869385	-5.249154121380	-2.997342129780
H	16.450758559559	-6.207758520632	-3.965459589857
C	18.975894750317	-6.484123785996	-5.082051758363
H	18.188042134494	-6.410566170001	-5.845311191104
H	19.787951158954	-7.088558989840	-5.491890844393
H	18.047970981075	-8.086597708556	-4.033288024018
C	20.666021997391	-4.742862414665	-2.347054967057
H	20.442507616914	-4.635557151959	-1.283659338556
H	21.433972126914	-3.997636053901	-2.593956747565
C	22.432264439516	-6.479404790675	-1.898302909611

H	23.212761320332	-5.731007863200	-2.094730898484
H	22.238486661673	-6.500594880785	-0.822615873487
H	22.820608917655	-7.462471349771	-2.173274686062
H	19.761622742981	-4.478747134743	-2.901592268704
H	21.390527502933	-6.192665796375	-3.744711296075
H	19.360199876707	-5.472182553763	-4.918818801492
H	16.873087918755	-6.722642598879	-2.319578677811

Complex 16

Si	-0.519618539741	0.043788979481	-1.054370778885
Cl	-1.395203498590	-1.731890249635	-1.634948404902
Cl	-1.959472121220	1.219665575117	-0.159278644248
C	0.119653773798	0.920133460664	-2.562370076943
C	0.210277340041	2.318321129222	-2.591141814092
C	0.591556389479	0.196376209829	-3.665596357285
C	0.758992471597	2.972983122025	-3.688104535670
H	-0.162807095073	2.899933352604	-1.755684256203
C	1.140345787556	0.850257989790	-4.763083105702
H	0.517510039274	-0.885192218096	-3.672067258329
C	1.225705498673	2.239254793799	-4.774729722244
H	0.817049581775	4.054911855077	-3.697690638824
H	1.495742098508	0.276814550447	-5.610957554569
H	1.649703920784	2.749354135902	-5.631580595812
C	0.789391628882	-0.335964349164	0.206787980139
H	1.558991770404	-0.974313949122	-0.233791479860
H	1.260878862830	0.589310355979	0.546599524875
H	0.362076421030	-0.848433693918	1.070536198562

Complex 17

Nb	19.719121907333	-8.073334178623	-1.621160779699
C	18.638614935686	-10.005088683787	-2.504208960980
C	17.621697158646	-9.046552749444	-2.246439240928
C	19.229257706571	-10.382163536955	-1.270635418533
C	17.605412559048	-8.802697891134	-0.851922247814
C	18.601992171573	-9.627252820833	-0.247145803779
C	19.981213061371	-5.700331728299	-1.508773034306
C	19.286341574119	-6.133810018888	-0.349742948214
C	21.278405952368	-6.270832388922	-1.469612565461
C	20.173223325484	-6.949423661653	0.416851227424
C	21.406021493251	-7.027004593969	-0.274523185705
H	19.019249032268	-7.278862233080	-3.004157583816
H	21.152576627997	-9.045661068508	-1.490658064069
H	19.603316196791	-5.066413863776	-2.292484627839
H	18.276619893382	-5.868684422528	-0.079655505820
H	19.945588456961	-7.415109352811	1.362498685636
H	22.026734397351	-6.172927366498	-2.239149728564
H	22.289901809743	-7.558513100898	0.035470943916

H	16.981095990523	-8.591231416577	-2.982515613282
H	18.946323306130	-10.372788519081	-3.469614445303
H	20.020328274505	-11.103055375403	-1.156422318189
H	18.820699128449	-9.685971211997	0.806934953448
H	16.945007714111	-8.124994938153	-0.334916911643
H	20.524839656440	-8.373359254125	-3.318106814082
Si	21.788218060023	-8.521656494998	-4.292638199318
C	21.720531281028	-8.967535972166	-6.115144163266
Cl	21.687882075680	-10.706677198590	-3.724250923468
Cl	21.206536339258	-6.374481079110	-4.793555121546
H	22.266997699553	-9.891988340986	-6.300789623176
H	20.677767284421	-9.123791457982	-6.405384461429
H	22.113166623708	-8.149458385896	-6.719165584667
C	23.419724014132	-8.112265180220	-3.444752193545
C	23.892110770549	-8.841187925258	-2.345596774575
C	24.206504331582	-7.049523907881	-3.911870871621
C	25.088929749733	-8.506807493175	-1.720422670814
H	23.315348518394	-9.682721268017	-1.984171699492
C	25.417249639496	-6.729141141054	-3.305396473621
H	23.857751013031	-6.457198177024	-4.748418373600
C	25.857495534780	-7.450318615712	-2.200295685117
H	25.428261821056	-9.080969523300	-0.865401765485
H	26.011524322013	-5.909257059119	-3.692157797180
H	26.794519241447	-7.194076273526	-1.719313250461

Complex 18

Nb	19.660127325600	-8.205111581700	-1.715134700368
C	18.671303600678	-10.251822180053	-2.430831962153
C	17.700195669624	-9.232838494266	-2.607544787047
C	18.939136638770	-10.387842511952	-1.041884024764
C	17.391642202764	-8.708687744867	-1.326556537268
C	18.157167297382	-9.426007860186	-0.357931917397
C	19.848637136654	-5.814497239559	-1.775639010205
C	19.161576655494	-6.176840530241	-0.592242689823
C	21.161850411774	-6.348263338375	-1.692325269556
C	20.057926678394	-6.927507334802	0.227820668767
C	21.301998239452	-7.015512855726	-0.449446596407
H	19.208983978205	-7.548721465758	-3.258715175956
H	21.001000210162	-9.225120987315	-1.273132657127
H	19.460719931995	-5.239106681384	-2.599224362735
H	18.142835050851	-5.922293585355	-0.346628044235
H	19.837474301048	-7.329732854947	1.203743731212
H	21.918169565497	-6.290021594677	-2.458075010492
H	22.195018398096	-7.507785376487	-0.104416882822
H	17.295445015300	-8.919391383421	-3.554356162748
H	19.139585790609	-10.798888159087	-3.232880311150
H	19.615378095418	-11.096746183386	-0.595046235542

H	18.131920083136	-9.272735182588	0.709105159224
H	16.682485777101	-7.923833154735	-1.117991653778
H	20.782285639601	-8.849904996048	-3.109630478349
Si	21.617568224189	-8.672209314682	-4.464985194647
C	22.528736384427	-10.039418775258	-5.373404723142
Cl	23.333154291560	-8.518812195833	-2.969937352355
Cl	19.679987905068	-9.252389503575	-5.473962697144
H	23.598169519276	-9.829685622750	-5.397239763207
H	22.386956146420	-10.980791976932	-4.835141301097
H	22.128222311217	-10.159799810670	-6.379772508609
C	21.687774564867	-6.885758881534	-5.058435919584
C	22.922918538943	-6.230649834597	-5.167474975388
C	20.535199851563	-6.169834706988	-5.407047070311
C	23.002716416669	-4.913314820000	-5.608584245938
H	23.828779883199	-6.753504842257	-4.887721374782
C	20.609048465169	-4.844402535298	-5.822700732923
H	19.573277152374	-6.663366196041	-5.357078685035
C	21.844457341167	-4.212000353877	-5.927534489282
H	23.970631743237	-4.432930070543	-5.693974737354
H	19.701341228889	-4.309051160394	-6.078162386232
H	21.904173958166	-3.181873871850	-6.259654762246

Complex 19

Nb	19.890287151715	-8.012541032920	-1.710310408841
C	18.941384042721	-10.020968247329	-2.587357758644
C	17.964259274324	-9.002755524020	-2.721438240033
C	19.175892073621	-10.246102510884	-1.205039947031
C	17.616173490479	-8.567136593936	-1.416321261706
C	18.362299806720	-9.343877576908	-0.478588085099
C	20.109636924720	-5.632247088036	-1.557774499074
C	19.377393319472	-6.088344273936	-0.435200464510
C	21.412650029488	-6.193841893345	-1.479128952587
C	20.237513560871	-6.917617800797	0.346674479300
C	21.501696814856	-6.968180660804	-0.293728548535
H	19.464227222295	-7.222582209465	-3.202821219930
H	21.224247881048	-9.059731024151	-1.334533674576
H	19.749229772700	-4.977685965218	-2.333117139581
H	18.354174111835	-5.839982240008	-0.202709225948
H	19.979331395216	-7.398963827464	1.276207429303
H	22.203828333875	-6.087716924042	-2.203469193959
H	22.376883513232	-7.502155568617	0.034059391105
H	17.578459547837	-8.629260813164	-3.654455049748
H	19.445212290873	-10.513845947657	-3.402823604424
H	19.854779522746	-10.976776107303	-0.798757823237
H	18.306592247256	-9.263125198372	0.595344470350
H	16.892410396487	-7.805328495626	-1.174198732762
H	21.095309516475	-8.170946064581	-3.173515327176

Si	22.084763732240	-8.995968764279	-4.125631762892
C	23.110950749902	-8.272727001720	-5.521486969811
Cl	20.240531242635	-8.975441478666	-5.467088482360
Cl	23.664842429737	-8.409300833263	-2.619718950997
H	22.807752043914	-8.703374037182	-6.475871575704
H	22.933127454447	-7.194836062870	-5.572308699918
H	24.172729790545	-8.433618307075	-5.336025288673
C	22.072836546641	-10.846196547689	-3.772498393608
C	21.674384359280	-11.748394721937	-4.769429088600
C	22.455749901995	-11.370791393560	-2.531275593689
C	21.660990189707	-13.120032498875	-4.535457236174
H	21.355481376046	-11.370128420655	-5.732474547329
C	22.417681866756	-12.739397730782	-2.285381346158
H	22.792890956756	-10.695651205802	-1.755432219535
C	22.022497840208	-13.619591077846	-3.288497704091
H	21.358817478325	-13.797097265070	-5.326151201646
H	22.710237029227	-13.119925395014	-1.313057639557
H	22.000757190772	-14.687016149131	-3.101000251962

Complex 20

Nb	19.737809102766	-8.090768908122	-1.692111314244
C	18.473500002591	-9.824466853767	-2.740662474768
C	17.657216213400	-8.662587135834	-2.740466917293
C	18.696696653914	-10.222071783793	-1.398414176141
C	17.396433910758	-8.320887854088	-1.391813638829
C	18.045871836627	-9.281510423017	-0.558207038397
C	20.418705709438	-5.804054944493	-1.649505862105
C	19.432000832468	-6.000016536629	-0.650095554063
C	21.546393224059	-6.589858865136	-1.298025945494
C	19.965666453141	-6.889123879672	0.331422145262
C	21.277844565000	-7.243322428784	-0.065068510592
H	19.541920549269	-7.309216970170	-3.231016112353
H	20.878733538210	-9.304073010856	-1.182477762825
H	20.341294262605	-5.188638660514	-2.529166213523
H	18.458783478980	-5.536906347170	-0.622318414108
H	19.463320513132	-7.219643904037	1.226464465840
H	22.437454079036	-6.686890881576	-1.896768205424
H	21.954036130759	-7.890061428768	0.467848900212
H	17.307948043415	-8.146112914967	-3.618594507513
H	18.886530497737	-10.306922019792	-3.611473018168
H	19.271798625461	-11.078300679513	-1.090259799121
H	18.020154771030	-9.305540799518	0.519544476527
H	16.802872302632	-7.487235721295	-1.051733623540
H	21.096452950011	-8.603449813062	-2.921523014358
Si	21.620969338963	-9.003158236910	-4.382425655809
C	23.405771477327	-9.345232988240	-4.854131838899
Cl	21.951348644280	-6.780977878666	-4.626528918054

Cl	21.456956128016	-11.091727517744	-3.482089534091
H	23.739057036121	-8.641758488115	-5.616855382202
H	24.038673924967	-9.216634670618	-3.971598715672
H	23.515883956614	-10.375412457533	-5.193088163121
C	20.225735021986	-9.194208561472	-5.633805948631
C	19.383729054677	-8.129261425879	-5.979534563366
C	20.003715637271	-10.431790336628	-6.254931071296
C	18.344663610587	-8.298603665166	-6.888762188305
H	19.552674721308	-7.157792170206	-5.533350209505
C	18.981698246754	-10.597838002270	-7.184652733514
H	20.627499262712	-11.277341957135	-5.993857001680
C	18.141524003716	-9.533989530617	-7.496800713435
H	17.701013866421	-7.460719122101	-7.132627434678
H	18.837234202594	-11.562278811271	-7.657868065959
H	17.337317529258	-9.665188194862	-8.211709006764

Complex 21

Nb	20.047305907838	-8.212151094218	-1.779317348749
C	18.745179796725	-9.977830553531	-2.717275238782
C	17.905062510336	-8.830521460195	-2.770807636308
C	18.907568471524	-10.321045892867	-1.346320184659
C	17.486325884378	-8.526627206209	-1.442522521383
C	18.100399916392	-9.438036440021	-0.570973625420
C	20.295901494652	-5.827023669661	-1.324655560720
C	19.400005598837	-6.162394669232	-0.267236241665
C	21.533041794320	-6.490217390757	-1.087287792736
C	20.049174872295	-7.073178312558	0.578172243495
C	21.360667488815	-7.299294679345	0.067625384830
H	20.080092459003	-7.426410790991	-3.374423139853
H	21.345499344808	-9.379478673103	-1.458645567120
H	20.100147682531	-5.129022022557	-2.122662123514
H	18.376319597339	-5.830358198697	-0.179740979344
H	19.617736492641	-7.562097584734	1.439033558940
H	22.437320939545	-6.391239134654	-1.664800888732
H	22.116910829083	-7.924745767968	0.513400524011
H	17.583827074702	-8.326702367196	-3.668308342207
H	19.151817370700	-10.523454662369	-3.553015226365
H	19.490102124826	-11.149140984192	-0.976918561543
H	18.027000933185	-9.436063822523	0.506242077904
H	16.858057296196	-7.696380566640	-1.156614264460
Si	21.630519578476	-9.029374693438	-3.519487435490
C	21.320576400464	-8.833173772481	-5.392691471431
Cl	22.025245275207	-11.202055368138	-3.548094543988
H	22.106977193010	-9.294449627988	-5.996700209738
H	20.367253418734	-9.302225243845	-5.655637238694
H	21.240085741290	-7.772864353601	-5.653384580113
C	23.391558873840	-8.356258892780	-3.264878993442

C	24.189812846319	-8.813438198967	-2.203497293607
C	23.893774801285	-7.286462911373	-4.021359808071
C	25.421399184042	-8.238457107966	-1.914111870994
H	23.826237240765	-9.637034438618	-1.597795277216
C	25.124378540980	-6.698894617732	-3.734973879909
H	23.308639842175	-6.898880782954	-4.849352338513
C	25.897300581543	-7.172742679388	-2.678588126966
H	26.015547955756	-8.618950934901	-1.088620084660
H	25.482089708709	-5.870549344500	-4.339416821874
H	26.855861576745	-6.718050627107	-2.451512240897

Complex 22

Nb	19.928867591851	-8.263370823848	-1.924800139404
C	18.901984313896	-10.289815880884	-2.585723810868
C	17.865413653243	-9.315976056779	-2.673339739459
C	19.187785650341	-10.474263867000	-1.204489192917
C	17.463712241593	-8.973987290270	-1.348711300531
C	18.273378583103	-9.681724245559	-0.450919001362
C	19.884775228384	-5.823871452476	-1.768716098187
C	19.013275432354	-6.161201938904	-0.692606180318
C	21.187894733041	-6.286979143625	-1.439391489191
C	19.754055846481	-6.856711250759	0.274253554826
C	21.093083566405	-6.981677082800	-0.201778861171
H	19.738201103633	-7.780828707053	-3.635801071939
H	21.376030427618	-9.217527710598	-1.584953164240
H	19.626135683981	-5.259657906063	-2.650715619168
H	17.953720415272	-5.955062001208	-0.655929313683
H	19.364840636233	-7.293491327383	1.181647920886
H	22.090048045168	-6.105488005589	-2.000170141491
H	21.915345227733	-7.445659740036	0.318464822162
H	17.409777291597	-8.960881093104	-3.583845271634
H	19.367213791631	-10.804889068719	-3.409992120560
H	19.921214091827	-11.151376116520	-0.798241252228
H	18.258977331172	-9.594034928572	0.625184109187
H	16.712469294797	-8.243214682424	-1.088958069444
Si	21.659711254233	-8.351823063563	-3.717872110122
C	21.520628316762	-9.708355191458	-5.050314156493
Cl	23.767432828524	-8.684382777847	-3.149641724982
H	22.325153159736	-9.627266762667	-5.786680896790
H	21.574180962566	-10.698906102231	-4.589958110908
H	20.560095514500	-9.625192025273	-5.569555792883
C	21.907089752113	-6.748547466735	-4.758436277965
C	23.074569335315	-5.972713935916	-4.760572772882
C	20.835164093280	-6.271114805106	-5.531618734101
C	23.168632367956	-4.786196039051	-5.485927521763
H	23.926625174520	-6.313393405210	-4.183364335918
C	20.922492873227	-5.095735280995	-6.271167037079

H	19.904605827373	-6.829908861507	-5.541287431868
C	22.093515325450	-4.341148933714	-6.248729652240
H	24.087630514253	-4.208348449714	-5.456338914958
H	20.073465289671	-4.763169220341	-6.861006784201
H	22.164511319122	-3.418820588501	-6.816180964102

Complex 23

Nb	20.008112946888	-7.986617653904	-1.717588144183
C	18.968245836321	-10.062844982569	-2.228443505910
C	17.961264445462	-9.084872911726	-2.465237201212
C	19.188478889326	-10.107362369214	-0.825614597973
C	17.507628975453	-8.597266501472	-1.204467270137
C	18.268075660124	-9.215603085471	-0.201736924266
C	19.938243424741	-5.548888893645	-1.819315646346
C	19.066653359581	-5.774561958705	-0.714035449759
C	21.245726345260	-5.965867355370	-1.443059659133
C	19.809654825990	-6.374875866097	0.313016453694
C	21.153165584136	-6.526371796370	-0.139001063203
H	19.887322406074	-7.638894075228	-3.456960074318
H	21.438033339362	-8.898346091730	-1.193043701314
H	19.672107735974	-5.076098670171	-2.751076759906
H	18.005055692779	-5.578114100175	-0.700972727176
H	19.422427522440	-6.719876994313	1.260053780545
H	22.153702434934	-5.844630819334	-2.010708741606
H	21.977893993497	-6.932079793267	0.423777583341
H	17.558360505576	-8.818489392928	-3.429065595920
H	19.465293248950	-10.667337793962	-2.969158098991
H	19.888352693324	-10.753511119202	-0.321179094099
H	18.206956542595	-9.016611632580	0.857928733860
H	16.761909328240	-7.830539455453	-1.056763786070
Si	21.955396840906	-8.487192207291	-3.189402771350
C	22.276716022085	-7.590491680306	-4.843446113949
Cl	23.955326816388	-8.148699425217	-2.315361953330
H	21.438102234517	-7.751118766812	-5.528814661809
H	22.344602337984	-6.512672542068	-4.665701178413
H	23.203156949538	-7.917451159756	-5.324024545005
C	22.076427551779	-10.333904900260	-3.631016374229
C	21.668589053461	-10.827808253706	-4.879416726718
C	22.440587454540	-11.282402754501	-2.661194055579
C	21.621631790458	-12.194228703390	-5.148651113102
H	21.375960828107	-10.130502673405	-5.658012734673
C	22.403763163342	-12.646807321892	-2.922152064639
H	22.756774253861	-10.931013920722	-1.684534965016
C	21.990570089043	-13.113333188600	-4.170452604510
H	21.298790557982	-12.542406914064	-6.125355172956
H	22.696390395297	-13.353274523718	-2.151142052399
H	21.955402543691	-14.178194261413	-4.375357022246

Complex 24

Nb	19.846610250670	-8.239591068340	-1.694218560667
C	18.262293235935	-9.909291496776	-2.347309405670
C	17.575653310553	-8.672211933372	-2.486548748590
C	18.477996187109	-10.122886243500	-0.957737660343
C	17.298282375021	-8.173831140459	-1.180424840183
C	17.837428433048	-9.067448012063	-0.243154520304
C	20.449961878739	-5.895087825803	-1.431566940258
C	19.608702716220	-6.026830906216	-0.287831079879
C	21.597660771561	-6.716515179237	-1.235705291120
C	20.195661744715	-6.955611772281	0.580945502187
C	21.409284639013	-7.413955514356	-0.010229404111
H	19.895325528847	-7.568651534810	-3.351186561607
H	21.014222283080	-9.527801414486	-1.384044957356
H	20.286971495602	-5.227995752215	-2.262915425582
H	18.652247715696	-5.544288512494	-0.152711953408
H	19.772263385258	-7.319048918592	1.505377399676
H	22.456301831953	-6.781111218305	-1.883455813734
H	22.102157548681	-8.112738963582	0.429178167118
H	17.266338228300	-8.230553340792	-3.420540891541
H	18.529182076243	-10.583718172596	-3.144259199973
H	18.969657470958	-10.979748127339	-0.526410222189
H	17.839550360029	-8.940915803672	0.829023489601
H	16.799674265414	-7.242039935750	-0.957650747768
Si	20.989475496284	-9.211695239172	-3.686497209808
C	22.591201161630	-8.386094372596	-4.309643139792
Cl	21.726543892870	-11.290898133177	-3.585412385175
H	22.397041493391	-7.338461024672	-4.562460225236
H	23.359608610463	-8.411553545764	-3.531846775170
H	22.981999483245	-8.893741661137	-5.195972156926
C	19.936123626724	-9.372644391769	-5.292655089118
C	19.429570096018	-8.205778072706	-5.889861351562
C	19.581359366875	-10.588092389540	-5.894158475634
C	18.625222424482	-8.244870982102	-7.024571259639
H	19.662252490845	-7.243966869846	-5.443738281772
C	18.764650438027	-10.640285986742	-7.022473282061
H	19.960501522239	-11.509757754525	-5.467568296839
C	18.283312315206	-9.468148413822	-7.597141260254
H	18.258269567743	-7.320099149167	-7.459881201266
H	18.505571061109	-11.601813718063	-7.455716009524
H	17.649053270212	-9.505574188153	-8.476954764517
